# Enterovirus A71 neuroinvasion: central nervous system entry, blood–brain barrier dynamics, and multistage constraints

**DOI:** 10.3389/fmicb.2026.1984969

**Published:** 2026-10-27

**Authors:** Yi Zhang, Zhi Li, Yu Gu, Bo Huang, Zhenzhuang Zou

**Affiliations:** 1 Zunyi Medical University, Zunyi, Guizhou, China; 2 Zunyi Medical and Pharmaceutical College, Zunyi, Guizhou, China

**Keywords:** blood–brain barrier, enterovirus A71, multistage progression constraints, neuroinvasion, neurotropism, retrograde axonal transport, systemic dissemination

## Abstract

Enterovirus A71 (EV-A71) is an important cause of severe neurological complications of hand, foot, and mouth disease in children. Most infections remain confined to peripheral sites, whereas only a small proportion progress to central nervous system (CNS) disease, and the mechanisms underlying this transition remain incompletely understood. Here, we reappraise EV-A71 neuropathogenesis as a multistage process comprising peripheral replication, systemic dissemination, CNS entry, establishment of productive CNS infection, and subsequent tissue injury. This distinction is important because these events are mechanistically connected but not interchangeable. Current evidence supports both neurovascular and peripheral neural routes of CNS entry, although the nature and strength of the supporting evidence differ. Human-derived blood–brain barrier (BBB) models demonstrate that EV-A71 and virus-associated carriers can engage and cross neurovascular interfaces, whereas temporal studies in animals, perturbation of axonal transport, and compartmentalized neuronal models provide stronger causal support for retrograde axonal transport once the virus has accessed peripheral nerves. These findings establish mechanistic feasibility, but the timing, necessity, and relative contribution of each route during natural infection in children remain unresolved. We further emphasize that BBB crossing is not synonymous with BBB disruption, and that CNS entry does not necessarily result in productive CNS infection. On this basis, we propose a multistage progression-constraint model, in which progression to severe neurological disease requires EV-A71 to overcome distinct constraints imposed by tissue context, viral properties, and host factors at successive stages. This framework provides a testable explanation for why most infections remain peripheral while only a minority progress to severe neurological disease, and highlights the need for longitudinal clinical studies and directly comparable experimental models to identify where disease trajectories first diverge.

## 1 Introduction

Enterovirus A71 (EV-A71) is an important cause of neurological complications associated with hand, foot, and mouth disease in children (Wei et al., 2024). Most infections are asymptomatic or mild, whereas a small proportion progress to central nervous system (CNS) disease, including brainstem encephalitis and acute flaccid paralysis (Chang et al., 2019; Wei et al., 2024). The mechanisms that allow EV-A71 to progress from peripheral infection to severe CNS disease remain incompletely understood.

Human pathological studies have localized EV-A71-associated lesions mainly to the spinal gray matter, brainstem, and selected deep nuclei, with viral antigen and RNA detected in neurons (Wong et al., 2008). These findings identify the regions affected by infection but do not establish how the virus initially reaches them. Studies of peripheral infection have shown that type III interferon responses can restrict viral replication in human intestinal models, while capsid variation can alter viral infectivity and receptor usage (Good et al., 2019; Aknouch et al., 2023). After the virus reaches the CNS, the establishment of productive infection also depends on the susceptibility of neural cells (Chooi et al., 2024). Thus, the factors influencing peripheral replication, CNS entry, and subsequent infection need to be considered separately.

Two principal routes have been proposed for EV-A71 entry into the CNS: blood-borne spread across neurovascular interfaces and spread along peripheral nerves (Tee et al., 2021). Studies using human-derived blood–brain barrier (BBB) models have shown that EV-A71-associated extracellular vesicles and infected immune cells can cross experimental endothelial barriers (Gu et al., 2023; Gaume et al., 2024). Animal studies and compartmentalized neuronal models provide evidence for retrograde axonal transport once EV-A71 gains access to peripheral nerves (Chen et al., 2007; Ohka et al., 2022). However, the timing and relative contributions of these routes during natural infection in children remain uncertain.

Viral interaction with brain endothelial cells, passage across the BBB, and disruption of barrier integrity are distinct events (Volle et al., 2015; Gu et al., 2023; Zhu et al., 2026). Experimental studies have demonstrated that EV-A71-associated extracellular vesicles can cross brain endothelial barriers without overt junctional disruption (Gu et al., 2023). Other studies have documented increased BBB permeability and endothelial junctional abnormalities during infection (Wang W. G. et al., 2025; Zhu et al., 2026). Thus, BBB damage observed during severe disease cannot, by itself, establish that barrier disruption was required for the initial entry of EV-A71 into the CNS.

This review examines EV-A71 neuropathogenesis from peripheral replication and systemic dissemination to CNS entry, productive infection, and subsequent tissue injury. Particular attention is given to neurovascular and peripheral neural entry and to the distinction between BBB crossing and barrier injury. We propose a multistage progression-constraint model in which viral, host, and tissue factors may limit disease progression at different stages. The model distinguishes systemic dissemination from CNS entry, CNS entry from productive infection, and productive infection from subsequent tissue injury. These distinctions provide a basis for examining stage-specific constraints through longitudinal studies and experimental interventions.

For this narrative review, literature was identified through PubMed searches, with the final search performed on 31 August 2026.

## 2 Peripheral replication and systemic dissemination

Human pathological studies and intestinal models support EV-A71 infection at peripheral mucosal sites, while clinical and animal studies provide evidence for spread beyond the initial sites of infection (Cheng et al., 2014; He et al., 2014; Lin et al., 2021; Tsang et al., 2021). Figure 1 summarizes the evidence for peripheral replication, viral release and transfer, systemic dissemination, and tissue-associated changes in viral populations, while distinguishing these events from candidate routes of CNS entry.

**Figure 1:**
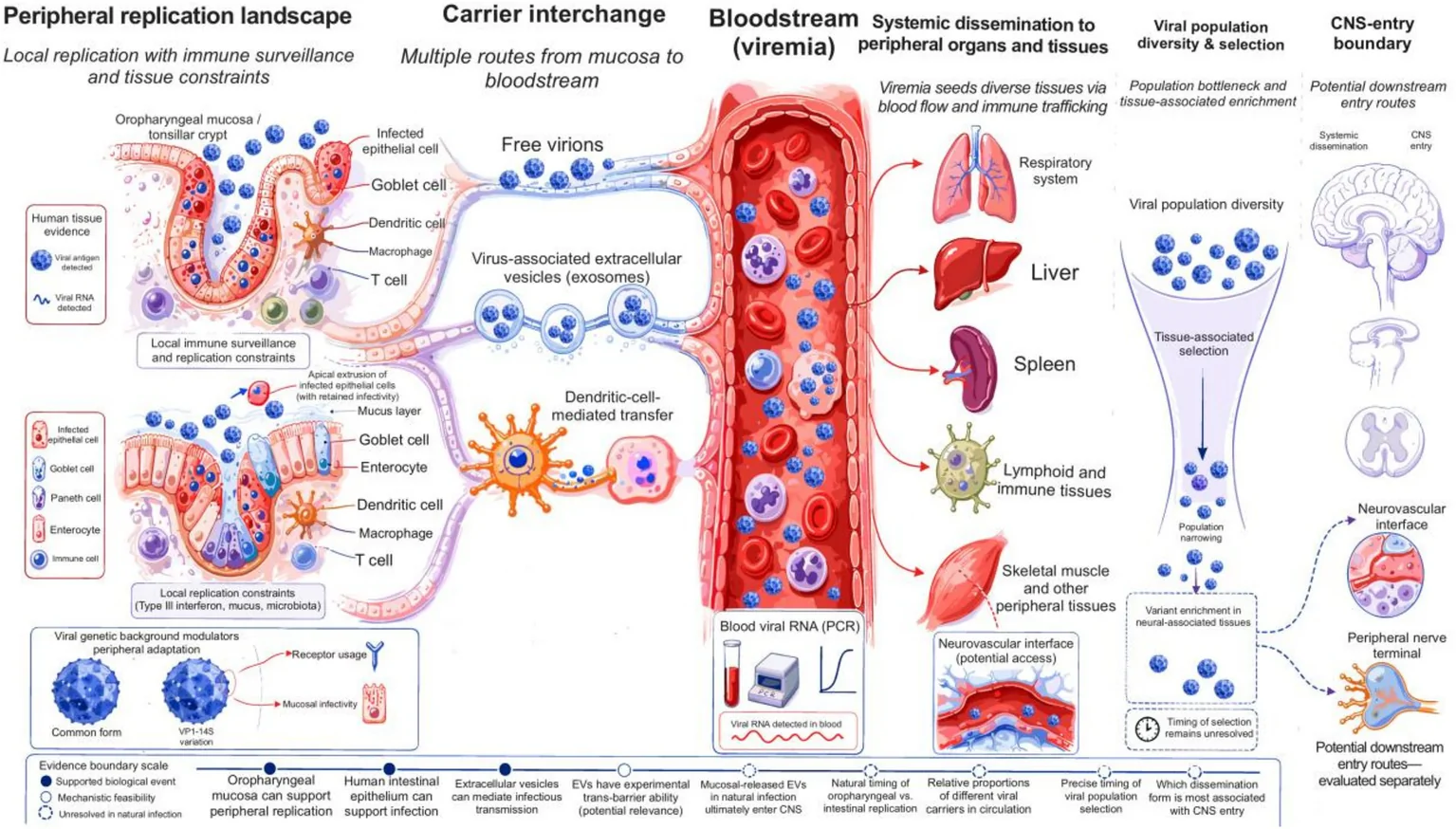
Peripheral replication, viral release and transfer, systemic dissemination, and candidate CNS entry routes during EV-A71 infection. BBB, blood–brain barrier; CNS, central nervous system; EVs, extracellular vesicles; EV-A71, enterovirus A71; PCR, polymerase chain reaction; VP1, viral protein 1. Human pathological studies implicate the oropharyngeal mucosa in peripheral EV-A71 infection, whereas human intestinal models demonstrate productive viral replication influenced by epithelial susceptibility, local immune responses, and viral genetic variation. Experimental studies have identified distinct forms of viral release and transfer, including free virions, virus-associated extracellular vesicles (EVs), and dendritic-cell-mediated trans-infection. In intestinal organoid models, infected epithelial cells can also be extruded toward the intestinal lumen while retaining infectivity; this process does not establish their entry into the bloodstream. Detection of EV-A71 RNA in blood provides evidence of systemic viral RNA dissemination but does not identify the physical form or infectivity of circulating viral material. Dissemination may expose peripheral organs and tissues to the virus, while tissue-associated changes in viral populations may occur at different stages of infection. The timing and anatomical location of viral selection remain unresolved.

Neurovascular and peripheral neural pathways are presented as candidate routes of CNS entry, although their relative contributions during natural infection in children remain unknown. The connecting pathways are schematic and do not imply that the same virus-associated carriers have been continuously traced from mucosal tissues through the circulation to the CNS during natural infection. The evidence boundary scale qualitatively distinguishes experimentally supported processes from mechanistic possibilities and steps that remain unresolved during natural infection.

### 2.1 Peripheral replication in the oropharynx and intestine

Autopsy studies of fatal EV-A71 encephalomyelitis detected viral antigen and/or RNA in the squamous epithelium lining tonsillar crypts, with viral signals showing a closer association with SCARB2 than with PSGL-1 expression (He et al., 2014). These findings implicate the tonsillar crypt epithelium as a site of peripheral infection, although autopsy samples cannot establish the sequence of early mucosal replication. In human small intestinal organoids, increasing viral loads and infectious virus titers demonstrated that the intestinal epithelium can support productive EV-A71 replication (Tsang et al., 2021).

Susceptibility to EV-A71 infection varies among intestinal epithelial cell populations and experimental models. In primary human intestinal epithelial monolayers, EV-A71 infected the epithelium from the apical surface and preferentially targeted goblet cells. Infection reduced goblet cell-derived mucin expression without overt impairment of epithelial barrier function, while type III interferon responses restricted viral replication (Good et al., 2019). In differentiated human intestinal organoids, more than 90% of VP1-positive cells expressed the enterocyte marker villin, and infection induced type III interferons and interferon-stimulated genes (Zhao et al., 2021). These studies identify both goblet cells and enterocytes as permissive intestinal cell populations and demonstrate the importance of local antiviral responses. Differences in the distribution of infected cell types should be interpreted in relation to the cellular composition and infection conditions of each model.

Viral capsid variation can further influence intestinal infection. In human fetal intestinal organoid-derived monolayers, a clinical isolate carrying glutamine at VP1 residue 145 (VP1-145Q) established infection following both apical and basolateral inoculation, whereas a VP1-145E isolate showed productive infection primarily after basolateral exposure. Site-directed mutants confirmed a replication advantage for VP1-145Q in this model (Aknouch et al., 2023). Pretreatment with low-molecular-weight heparin significantly reduced infection by VP1-145Q viruses, supporting a role for heparan sulfate proteoglycan (HSPG)-associated attachment (Aknouch et al., 2023). These findings connect capsid variation, epithelial polarity, and attachment-dependent infectivity to the efficiency of peripheral viral amplification.

### 2.2 Vesicle-associated release and cell-associated viral transfer

EV-A71 can leave infected epithelial cells through mechanisms other than lytic release of free virions. In differentiated human intestinal epithelial cells and intestinal organoids, infection promotes the release of exosome-enriched fractions containing viral components that can establish infection in recipient cells (Huang et al., 2020). In RD cells, the viral nonstructural protein 3A interacts with VPS25, a component of the ESCRT-II complex, to promote exosome biogenesis. Silencing VPS25 reduces vesicle release and viral replication at later stages of infection (Ruan et al., 2022). These findings provide evidence for vesicle-associated viral release and identify a host pathway involved in regulating this process.

Infected cells and virus-bearing immune cells provide other forms of transfer. In human colonic organoids, EV-A71-infected epithelial cells are extruded from the apical surface through a process dependent on mechanosensitive ion channel activity. The extruded cells remain infectious and can initiate infection in recipient cells (Moshiri et al., 2023). This mechanism describes release toward the intestinal lumen rather than entry of intact epithelial cells into the bloodstream. Separately, human monocyte-derived dendritic cells can transfer captured EV-A71 to susceptible cells without supporting productive viral replication. Inhibition of this trans-infection by soluble heparin implicates heparan sulfate-associated binding in viral transfer (Helgers et al., 2022).

Clinical evidence for vesicle-associated viral material in the circulation remains limited. EV-A71 RNA was detected in exosome-enriched plasma fractions from five children with severe hand, foot, and mouth disease complicated by viral encephalitis (Gu et al., 2020). This observation does not establish the infectivity or tissue origin of the associated material. Experimental studies have separately demonstrated the capacity of EV-A71-associated small extracellular vesicles to cross BBB models (Gu et al., 2023). Together, these findings support individual steps in a possible dissemination pathway, but do not trace the same vesicle population from the intestinal epithelium to the CNS during natural infection.

### 2.3 Systemic dissemination and evidence from viremia

Detection of EV-A71 RNA in blood provides clinical evidence of systemic viral RNA dissemination, although PCR positivity alone does not establish the presence of circulating infectious virions. In a multicenter cohort of 224 children, viral RNA was detected in blood from 59 patients (26%), with 68% of positive cases identified within the first 3 days after symptom onset (Cheng et al., 2014). The presence of PCR-detected viremia was not associated with complicated disease in the cohort overall. Among viremic patients, however, those sampled after day 3 had a higher frequency of severe complications than those sampled earlier (26% versus 5%). Because blood was collected only once from each patient, these findings do not establish the duration of viremia in individual children or its temporal relationship to CNS entry.

Animal studies demonstrate that peripheral exposure can be followed by infection of distant tissues, including the CNS. In rhesus macaques, viral distribution, viremia, and CNS involvement differed following intravenous, respiratory, and digestive inoculation (Zhang et al., 2011). Following oral inoculation of hSCARB2-transgenic mice with mouse-adapted EV-A71/MP4, bioluminescence imaging and viral antigen staining demonstrated viral spread to multiple tissues, including the brain and spinal cord (Lin et al., 2021). These findings establish multiorgan involvement after peripheral inoculation without identifying a single anatomical route of CNS entry.

EV-A71 populations may also change as infection involves different tissues. Deep sequencing of samples from a fatal human case revealed differences in viral haplotypes across respiratory, intestinal, skin, and CNS-associated specimens, with VP1-31G enriched in neural tissues (Huang et al., 2017). In cell-based assays, VP1-31G showed a replication advantage in SK-N-SH neuroblastoma cells, whereas VP1-31D replicated more efficiently in DLD-1 colorectal cells. Cell-type-dependent differences in viral RNA uncoating provided a possible mechanism for these replication phenotypes (Huang et al., 2017). These observations are consistent with tissue-associated selection, but the clinical samples cannot determine whether enrichment of VP1-31G occurred before CNS entry or during replication within neural tissue. Capsid-dependent attachment also does not translate directly into pathogenicity. In B-cell-depleted mice, VP1-L97R increased heparan sulfate binding and enhanced pathogenicity, whereas the additional VP1-E167G substitution further increased binding but attenuated the virus in both immunocompetent and B-cell-depleted mice (Weng et al., 2023). Together, these findings distinguish tissue-associated viral fitness and attachment phenotypes from the ability to cross a CNS entry interface (Figure 1).

## 3 CNS entry: comparing evidence for neurovascular and peripheral neural routes

Two principal categories of candidate pathways have been investigated for EV-A71 entry into the CNS: blood-borne access across CNS interfaces and spread along peripheral nerves (Figure 2). Extracellular vesicles and virus-bearing leukocytes represent potential carriers within the blood-borne pathway rather than additional anatomical routes (Table 1). The following sections distinguish endothelial infection, experimental barrier crossing, and retrograde axonal transport according to the processes directly examined in each model.

**Figure 2:**
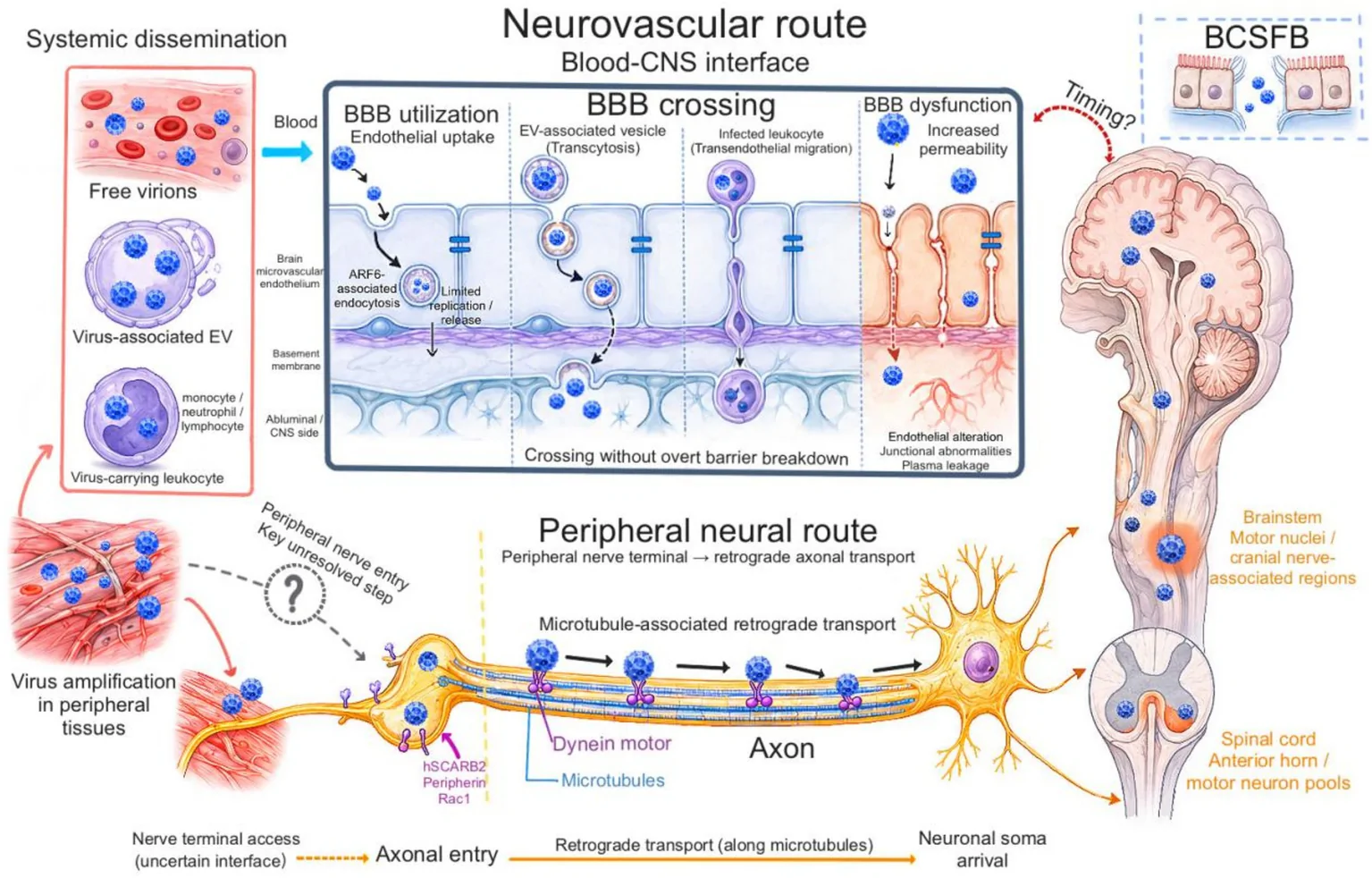
Experimental evidence for neurovascular and peripheral neural routes of EV-A71 entry into the CNS. ARF6, ADP-ribosylation factor 6; BBB, blood–brain barrier; BCSFB, blood–cerebrospinal fluid barrier; CNS, central nervous system; EVs, extracellular vesicles; EV-A71, enterovirus A71; hSCARB2, human scavenger receptor class B member 2. Following systemic dissemination, EV-A71 may encounter blood–CNS interfaces as free virions, virus-associated extracellular vesicles (EVs), or virus-bearing leukocytes. Experimental blood–brain barrier (BBB) models distinguish three processes: endothelial engagement and uptake, passage across the endothelial barrier, and subsequent barrier dysfunction. ARF6-associated endocytosis contributes to EV-A71 uptake by brain microvascular endothelial cells (Zhu et al., 2023). EV-A71-associated EVs can cross experimental endothelial barriers, while virus-bearing leukocytes can migrate across BBB models without requiring overt barrier breakdown (Gu et al., 2023; Gaume et al., 2024). These findings support distinct mechanisms of experimental barrier crossing, but endothelial uptake alone does not establish CNS entry. Increased permeability and junctional abnormalities are depicted separately because their temporal relationship to initial viral crossing remains unresolved during natural infection. The blood–cerebrospinal fluid barrier (BCSFB) is shown as a separate candidate interface; infection and polarized viral recovery in BCSFB-associated models do not establish its contribution to CNS entry in children (Hügel et al., 2026). After gaining access to peripheral nerve endings, EV-A71 can undergo retrograde axonal transport toward neuronal somata in experimental systems. Animal infection studies and compartmentalized motor neuron models provide evidence for neural spread under defined conditions, including hSCARB2-associated transport and subsequent productive infection of neuronal cell bodies (Chen et al., 2007; Ohka et al., 2022). Peripherin and Rac1 have also been implicated in neuronal entry and neuroinvasion-related processes, respectively, but their depiction does not imply that they form a single molecular complex with hSCARB2 (Lim et al., 2021). Retrograde movement is illustrated along microtubules, with dynein involvement presented as a candidate mechanism rather than a quantitatively established transport process for EV-A71. How the virus reaches peripheral nerve endings after natural mucosal infection, and the relative contribution of neural versus blood-borne entry during infection in children, remain unresolved.

**Table 1:** Evidence hierarchy and unresolved steps for candidate CNS entry routes and carrier mechanisms of EV-A71.

Candidate route or carrier mechanism	Sequence	Evidence base	Supported steps	Key gaps	Relevance to natural infection
Neurovascular route (BBB; BCSFB as a candidate interface)	Peripheral infection → circulation → blood–CNS interface → potential CNS access	Mechanistic: cell/barrier models; *in vivo*: limited; clinical route: unresolved	Brain endothelial engagement; experimental BBB crossing by virus-associated vesicles; transmigration of virus-bearing leukocytes; genotype-dependent infection of BCSFB epithelial models	Relative contribution in vivo; whether BCSFB infection leads to CNS entry; timing of barrier crossing versus injury	Blood-borne CNS access is experimentally feasible, but its contribution during natural infection remains unknown
Peripheral neural route	Peripheral infection → nerve-ending access → retrograde axonal transport → CNS	Mechanistic: neuronal/transport models; in vivo: animal support; clinical route: unresolved	Axonal entry and retrograde transport; anatomically associated spread; effects of transport-related interventions	Access to nerve endings after natural mucosal infection; relative contribution in children	Supports neural spread after nerve access, but does not establish predominance during natural infection
Vesicle-associated transport (within the blood-borne route)	Virus-associated vesicles → endothelial uptake → experimental barrier passage	Mechanistic: vesicle transmission and BBB models; in vivo contribution: unknown	Vesicle-associated transmission; experimental BBB crossing	Identity and infectivity of circulating vesicle-associated virus; contribution to CNS entry	A potential blood-borne carrier mechanism, not an independent anatomical route
Leukocyte-associated transport (within the blood-borne route)	Virus-bearing leukocytes → blood–CNS interface → experimental transmigration	Mechanistic: leukocyte migration and barrier models; in vivo contribution: unresolved	Association of EV-A71 with immune cells; experimental transmigration of virus-bearing leukocytes	Principal carrier-cell types in vivo; delivery of infectious virus; contribution to productive CNS infection	A potential blood-borne carrier mechanism; its contribution during natural infection remains unknown

The first two rows describe candidate anatomical routes of CNS entry. The last two describe carrier mechanisms within blood-borne access and should not be interpreted as additional anatomical routes.

BBB, blood–brain barrier; BCSFB, blood–cerebrospinal fluid barrier; CNS, central nervous system; EV-A71, enterovirus A71.

### 3.1 Neurovascular route: distinguishing BBB engagement, crossing, and disruption

Brain microvascular endothelial cells provide a potential interface for circulating EV-A71, but endothelial infection and passage across the blood–brain barrier (BBB) are distinct experimental endpoints. In polarized hCMEC/D3 cultures, EV-A71 replicated its genome, although viral antigen was detected in relatively few cells and infectious virus was released at very low levels from both sides of the endothelial layer without detectable barrier dysfunction (Volle et al., 2015). Viral entry into human brain microvascular endothelial cells involves ARF6-dependent endocytosis, with ARF1 also contributing to this process (Zhu et al., 2023). In primary brain microvascular endothelial cells from tree shrews, antibody blockade of SCARB2 or ANXA2 reduced viral RNA and protein levels, supporting their involvement in cellular infection (Wang W. G. et al., 2025). These studies identify mechanisms of endothelial susceptibility and uptake, rather than establishing efficient transport across an intact BBB.

More direct evidence for experimental BBB crossing comes from virus-associated extracellular vesicles. EV-A71-associated small extracellular vesicles were taken up by brain microvascular endothelial cells and released on the abluminal side of an *in vitro* BBB model, where they infected astrocytes (Gu et al., 2023). This transport occurred without overt tight-junction disruption at early time points, whereas later junctional damage was associated with reduced PI3K/Akt signaling (Gu et al., 2023). Microvesicles carrying EV-A71 virions also crossed experimental BBB models. Their uptake involved a non-clathrin-dependent pathway, and damaged mitochondrial cargo promoted barrier crossing and contributed to cellular injury (Tian et al., 2025). These studies demonstrate vesicle-associated transport under experimental conditions, with different relationships between barrier crossing and junctional damage.

Leukocyte-associated transport provides another mechanism of experimental crossing. In a human BBB model containing brain-like endothelial cells and pericytes, passage of cell-free EV-A71 was strongly restricted, whereas infected immune cells, including monocytes, neutrophils, and NK/T cells, migrated across the endothelial layer (Gaume et al., 2024). These results support infected leukocytes as potential carriers across the neurovascular interface, distinct from the transcellular transport of virus-associated vesicles.

The BBB dysfunction should be considered separately from the initial entry mechanisms. Recombinant VP1 increased endothelial permeability, reduced claudin-5 expression, and upregulated vimentin through TGF-β/Smad3 and NF-κB signaling in experimental systems (Wang et al., 2020). In EV-A71-infected tree shrews, increased BBB permeability was accompanied by reduced expression of claudin-5 and JAM-A (Wang W. G. et al., 2025). Infection-associated barrier injury has also been linked to C5a–C5aR1-dependent neutrophil recruitment and neutrophil extracellular trap formation (Zhu et al., 2026). Their involvement in BBB injury does not establish that barrier disruption preceded the first entry of EV-A71 into the CNS.

The blood–cerebrospinal fluid barrier (BCSFB) is a distinct candidate interface for blood-borne CNS access. Human choroid plexus papilloma (HIBCPP) cells and brain microvascular endothelial cell models showed genotype-dependent differences in EV-A71 infection. Genotype B5 produced relatively high infection levels in HIBCPP cells and showed preferential recovery of viral RNA from the cerebrospinal fluid-facing compartment (Hügel et al., 2026). These findings provide evidence for infection and polarized viral recovery in a BCSFB-associated model. They do not, however, establish the contribution of passage across the intact choroid plexus barrier to CNS entry during natural infection.

### 3.2 Peripheral neural route: from anatomical correlation to causal support

Human neuropathological and imaging studies provide anatomical evidence consistent with peripheral neural spread. Viral antigen has been detected in neuronal cell bodies and processes within the spinal cord and brainstem, as well as in spinal nerve roots and intestinal autonomic ganglia. Magnetic resonance imaging has also revealed segmental patterns of spinal cord and brainstem involvement (Xing et al., 2016; Li et al., 2019). These findings are compatible with spread along peripheral neural pathways, although anatomical distribution alone does not establish the direction of viral transport.

Animal experiments provide stronger evidence for the direction of spread. Following intramuscular inoculation, the initial distribution of spinal cord infection depended on the inoculated limb, and colchicine treatment reduced viral titers in the spinal cord and brain. However, sciatic nerve transection did not significantly reduce CNS infection in this mouse-adapted virus model (Chen et al., 2007). Other studies detected early viral signals in ipsilateral spinal anterior horn neurons and adjacent axons after intramuscular inoculation, or in ipsilateral cranial nerves and corresponding brainstem nuclei after jaw and facial muscle inoculation (Ong et al., 2008; Tan et al., 2014). In adult hSCARB2-transgenic mice infected with non-mouse-adapted EV-A71, virus was detected in the spinal cord of two of four sham-operated mice but was not detected in the sciatic nerve-transected group at 72 h after intramuscular inoculation (Ohka et al., 2022). These findings support neural spread under defined experimental conditions, while showing that the effects of nerve interruption differ between models.

Cellular studies identify mechanisms involved in neuronal infection and subsequent spread. Peripherin colocalized with EV-A71 at neuromuscular junctions in infected mice, and surface-expressed peripherin facilitated viral entry into neuronal cell models; Rac1 was also implicated in EV-A71 neuroinvasion-related processes (Lim et al., 2021). In compartmentalized motor neuron cultures, inoculation of the axonal compartment led to subsequent detection of infectious EV-A71 in the somatic compartment and viral replication in neuronal cell bodies. These findings depended on hSCARB2 expression, and anti-hSCARB2 treatment inhibited infection after axonal inoculation (Ohka et al., 2022). The experiments support spread from the axonal compartment to neuronal somata, where productive infection can be established, but do not quantify axonal transport efficiency independently of viral entry and replication.

The efficiency of retrograde transport is a separate question from its experimental demonstration. In a poliovirus study using genetically marked viral populations, only 28% of the viral population members detected in the lower sciatic nerve were retained in the upper sciatic nerve, indicating a substantial transport bottleneck (Lancaster and Pfeiffer, 2010). This measure of population member retention cannot be directly compared with viral titers recovered after axonal inoculation in EV-A71 neuronal models. Although both viruses can undergo retrograde axonal transport, available studies do not establish that EV-A71 is transported more efficiently than poliovirus (Ohka et al., 2022). Moreover, intramuscular inoculation places virus close to peripheral nerve endings, whereas the route by which EV-A71 reaches these endings after natural oropharyngeal or intestinal infection remains to be established. Retrograde transport is therefore an experimentally supported mechanism of neural spread, but its relative contribution to CNS entry during natural infection in children remains unresolved (Figure 2).

## 4 Establishment of CNS infection and secondary tissue injury

Following CNS entry, EV-A71 must establish productive infection in susceptible cells before local viral amplification can occur. Attachment, uncoating, and RNA replication depend on distinct host factors, while subsequent tissue injury reflects both viral replication and local immune responses.

### 4.1 CNS exposure is not equivalent to productive infection

Productive infection requires compatible mechanisms of attachment, internalization, and uncoating. SCARB2 is a major functional receptor for EV-A71, but its role should be distinguished from that of initial attachment factors. In the susceptible cell lines examined, SCARB2 knockout blocked productive infection without substantially reducing viral attachment, consistent with a critical role in acid-dependent uncoating within late endosomes and lysosomes (Yamayoshi et al., 2009; Nishimura et al., 2024). Cell surface attachment can involve other molecules, including PSGL-1 in a strain-dependent manner (Nishimura et al., 2013). SLC35B2 further supports infection by regulating both heparan sulfate sulfation and SCARB2 tyrosine sulfation, thereby influencing viral attachment and subsequent entry processes (Guo et al., 2022). These findings show that cellular attachment and productive viral entry impose different requirements.

Host factors also regulate the steps that follow viral uptake. SPNS1 interacts with EV-A71 VP1 and SCARB2 in endolysosomal compartments and facilitates release of the viral pocket factor during uncoating. SPNS1 deficiency inhibited infection but had little effect on replication of viral RNA introduced directly into the cytoplasm, supporting a function before cytoplasmic RNA replication (Fu et al., 2025). ROCK1 and VEGFR2 have also been identified as host factors supporting EV-A71 replication in non-CNS experimental systems, although their roles in infected neural cells remain to be established (Zhao et al., 2022; Zhao et al., 2024). In contrast, AIMP2 restricts replication by recruiting the E3 ligase SMURF2 to promote degradation of the viral 3D polymerase (Ren et al., 2024). These studies distinguish host functions that facilitate viral infection from intracellular mechanisms that limit replication.

The consequences of CNS exposure also depend on the neural cell populations involved. In human pluripotent stem cell-derived neuronal cultures, motor neurons exhibited relatively high viral RNA loads and supported productive EV-A71 infection (Chooi et al., 2024). By contrast, direct intracerebral inoculation in mice revealed restricted interneuronal spread within the brain parenchyma despite SCARB2 expression (Hamidi et al., 2025). Thus, susceptibility of individual neural cells does not necessarily predict extensive viral spread within CNS tissue. Differences in viral tropism across experimental systems should also be considered when interpreting the relative involvement of neurons and glial cells.

### 4.2 Neural injury as a combined consequence of viral replication and local host responses

Productive EV-A71 infection can directly injure susceptible neurons. In human stem-cell-derived neuronal models, infected motor neurons showed reduced expression of ferritin-associated genes, increased labile iron, and enhanced lipid peroxidation. Treatment with the iron chelator deferoxamine improved mitochondrial function and neuronal survival, supporting a contribution of ferroptosis-associated injury in this model (Chooi et al., 2024).

Glial cells can amplify injury through local inflammatory signaling. In hSCARB2 knock-in mice, viral replication was detected predominantly in astrocytes, with more limited involvement of neurons and microglia, and was accompanied by gliosis (Jin et al., 2021). In separate experimental models, EV-A71 infection activated astrocytic TLR7 signaling and increased IL-6 production. TLR7 deficiency or knockdown reduced infection-associated injury, while IL-6 neutralization attenuated neurological disease in mice (Luo et al., 2019). Complement signaling provides another intervention-supported mechanism. C5a–C5aR1 activation in astrocytes increased CXCL1 production through p38 MAPK-associated signaling, whereas C5aR1 blockade or CXCL1 neutralization reduced experimental neuropathology (Zhu et al., 2025). These findings identify distinct astrocytic pathways that can contribute to tissue injury after infection.

Myeloid cell responses provide additional inflammatory mechanisms. In human THP-1-derived macrophages, EV-A71-induced IL-1β production involved NLRP3 and RIG-I inflammasome pathways, with contributions from TLR3, TLR8, caspase-1, and caspase-8 (Huang et al., 2022). In neonatal mice, TLR2–NF-κB–NLRP3 signaling contributed to microglial activation and neuroinflammation; inhibition of TLR2 or NLRP3 reduced neurological injury (Li et al., 2026). These studies identify inflammatory pathways in different myeloid systems rather than establishing a single mechanism shared by peripheral macrophages and CNS-resident microglia.

Clinical studies have also identified altered innate and adaptive immune profiles in children with severe EV-A71 infection, including changes in myeloid and T cell responses (Li et al., 2021; Xu et al., 2023; Wang C. et al., 2025; Wang M. et al., 2025). These observations provide clinical context for immune dysregulation, while the experimental findings above more directly identify mechanisms of local neural injury.

### 4.3 Stage-dependent roles of the BBB: from restrictive interface to target of inflammatory injury

The mechanisms that permit virus-associated material to cross experimental neurovascular barriers differ from those responsible for BBB dysfunction during infection. Figure 3 integrates these processes into a stage-dependent working framework. Experimental evidence for barrier injury includes HMGB1-associated changes in endothelial junctions and complement-dependent recruitment and activation of neutrophils (You et al., 2023; Zhu et al., 2026).

**Figure 3:**
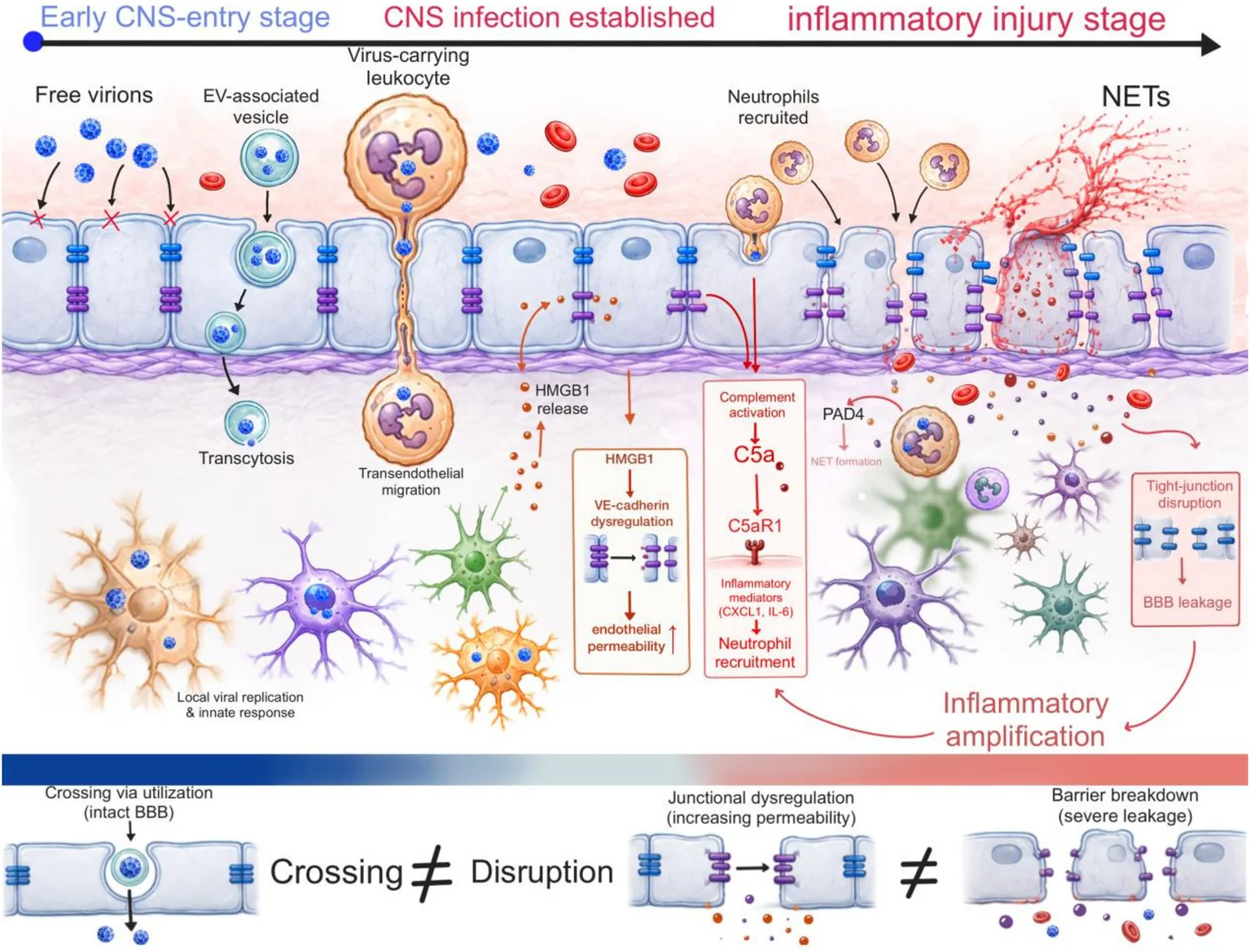
Distinct roles of the blood–brain barrier during EV-A71 neuroinvasion and infection-associated inflammatory injury. BBB, blood–brain barrier; C5aR1, C5a receptor 1; EV-A71, enterovirus A71; HMGB1, high mobility group box 1; NETs, neutrophil extracellular traps; PAD4, peptidyl arginine deiminase 4. Experimental BBB models demonstrate restricted passage of free virions, whereas EV-A71-associated extracellular vesicles and virus-bearing leukocytes can cross endothelial barriers through transcellular transport and cellular migration, respectively (Gu et al., 2023; Gaume et al., 2024). BBB injury involves distinct mechanisms, including HMGB1-associated VE-cadherin phosphorylation at Tyr685 and increased endothelial permeability (You et al., 2023), as well as C5a–C5aR1-dependent neutrophil recruitment and PAD4-associated neutrophil extracellular trap formation (Zhu et al., 2026). The diagram distinguishes carrier-mediated crossing of a relatively intact BBB, junctional dysregulation, and severe barrier breakdown. These states should not be interpreted as obligatory consecutive stages, as their temporal relationship during natural infection in children remains unresolved.

EV-A71 infection induces extracellular release of high mobility group box 1 (HMGB1), which can impair endothelial junctional regulation. In human brain microvascular endothelial cell models, HMGB1 increased VE-cadherin phosphorylation at Tyr685 and endothelial permeability. Neutralization of HMGB1 attenuated both changes, providing intervention-level evidence for its contribution to barrier dysfunction (You et al., 2023).

Complement-dependent inflammatory responses provide another mechanism of BBB injury. In EV-A71-infected mice, C5a–C5aR1 signaling promoted neutrophil accumulation and neutrophil extracellular trap (NET) formation. C5aR1 deficiency reduced BBB leakage and preserved tight-junction protein expression, while neutrophil-specific PAD4 deletion or pharmacological NET inhibition attenuated barrier injury and neuroinflammation (Zhu et al., 2026). These findings identify a neutrophil-associated mechanism of vascular damage that is mechanistically distinct from astrocytic C5a–C5aR1–CXCL1 signaling.

Together, these studies provide mechanistic evidence for inflammatory BBB destabilization during EV-A71 disease. However, the timing of these injury processes relative to the initial CNS entry event has not been established in naturally infected children. The stage-dependent sequence illustrated in Figure 3 therefore represents an integration of findings from different experimental systems rather than a fixed temporal course demonstrated by longitudinal human observations.

## 5 Multistage progression constraints: a testable working model

The evidence reviewed above can be integrated into a working model comprising five related processes: peripheral replication, systemic dissemination, CNS entry, establishment of productive CNS infection, and infection-associated tissue injury (Figure 4). We use the term “multistage progression constraints” to describe the viral, tissue, and host conditions that influence progression between these processes (Table 2). The stages are analytical distinctions rather than an obligatory sequence detectable in every patient. In the proposed peripheral neural route, access to nerve endings could occur without a detectable viremic phase, although this possibility remains unconfirmed during natural infection. Completion of one process therefore creates an opportunity for subsequent progression without establishing that the next process has occurred.

**Figure 4:**
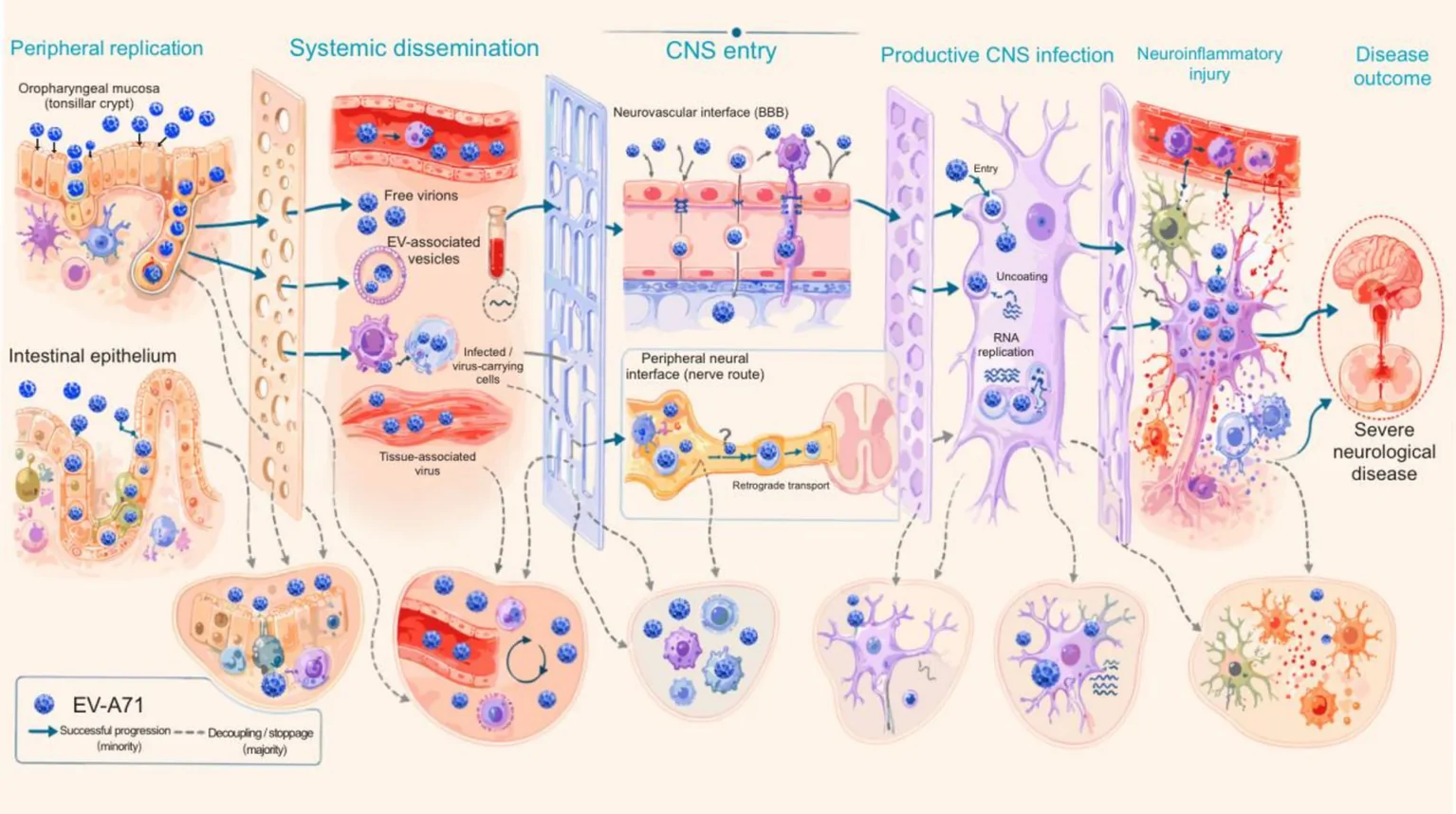
Multistage progression-constraint model of EV-A71 neurological disease. BBB, blood–brain barrier; CNS, central nervous system; EV-A71, enterovirus A71. The model distinguishes five related processes: peripheral replication, systemic dissemination, CNS entry, establishment of productive CNS infection, and neuroinflammation and tissue injury, followed by clinical outcomes. Each transition is influenced by viral, tissue, and host factors, and completion of one process does not guarantee progression to the next. Neurovascular and peripheral neural pathways represent candidate routes of CNS entry, although their relative contributions during natural infection remain unresolved. Solid arrows indicate potential progression between stages, whereas dashed arrows represent possible arrest or decoupling without implying specific probabilities. The diagram represents a testable conceptual framework rather than an obligatory temporal sequence established in naturally infected children.

**Table 2:** Stage-specific constraints during progression of EV-A71 neurological disease.

Stage	Transition	Key constraints	Evidence	Consequence	Key question
Peripheral replication	Mucosal exposure → local amplification	Tissue permissiveness; cell composition; mucosal immunity; viral adaptation	Human samples; mucosal models; organoids	Provides virus for subsequent dissemination	Dominant early replication niche?
Systemic dissemination	Local replication → circulation/distant tissues	Release mode; circulatory stability; immune clearance; carrier selection	Viremia; animal models; multiorgan analyses	Creates access to potential CNS entry sites	What is the predominant unit of dissemination?
CNS entry	Peripheral exposure → CNS exposure	BBB restriction; neurovascular interface; nerve-ending accessibility	BBB models; animal tracing; axonal transport	Provides an opportunity for CNS localization	What determines route selection?
Productive CNS infection	CNS exposure → productive infection in susceptible CNS cells	Receptor compatibility; uncoating; replication competence; antiviral restriction	Neuronal models; receptor studies; replication studies	Establishes foci of productive infection	Why does some CNS exposure fail to establish infection?
Neuroinflammation and tissue injury	Viral infection → pathological injury	Viral injury to susceptible neural cells; inflammatory amplification; complement; HMGB1; NETs; BBB destabilization	Animal interventions; immune studies; BBB models	Shapes disease severity	What governs the shift from protection to injury?

Clinical and experimental observations illustrate why these processes should be assessed separately. In a pediatric cohort, PCR-detected viremia was not associated with complicated disease in the population overall, although severe complications were more frequent among viremic patients sampled after day 3 than among those sampled earlier. Thus, blood viral RNA alone is an insufficient marker of clinical progression, and the study does not establish the temporal relationship between viremia and CNS entry (Cheng et al., 2014). At a later stage of the proposed framework, direct intracerebral inoculation in mice revealed restricted interneuronal spread despite SCARB2 expression, indicating that CNS exposure does not necessarily lead to extensive propagation within neural tissue (Hamidi et al., 2025). These findings concern different transitions and do not establish the complete sequence of disease progression within individual naturally infected children.

The model generates two related predictions. First, measurements representing adjacent stages should not invariably change together. For example, systemic viral dissemination may occur without demonstrable productive CNS infection, while the extent of BBB injury may differ between infections with comparable CNS viral burdens. Second, interventions directed at different processes should yield distinguishable effects on stage-specific readouts, such as circulating viral burden, CNS viral replication, and BBB permeability, when viral strain, inoculation conditions, and observation time are controlled. An intervention that reduces inflammatory BBB injury after CNS infection has been established, for instance, need not prevent an earlier CNS entry event. Longitudinal measurements and appropriately timed interventions could test these predictions; failure to observe the expected distinctions under comparable conditions would require the framework to be revised.

A progression constraint is not synonymous with a population-genetic transmission bottleneck. Tissue-associated enrichment of EV-A71 variants may reflect selection during dissemination or replication within an infected tissue, but changes in viral diversity do not identify the anatomical transition at which selection occurred (Huang et al., 2017). The framework also does not assume that individual host factors act exclusively at one stage or that all infections follow a fixed temporal course. Its principal value is to make the relationships between stages experimentally examinable.

## 6 Discussion

The central challenge in EV-A71 neuroinvasion research is to determine how mechanisms demonstrated in different experimental systems contribute to disease during natural infection. Three questions are particularly important: how model design influences inference about CNS entry routes, what evidence can distinguish those routes, and how the multistage progression-constraint model can be evaluated through longitudinal observation and intervention.

### 6.1 How model dependence shapes inference about transmission routes

The experimental starting point determines which segment of viral transmission can be examined. *In vitro* BBB models introduce virus or virus-associated carriers at the vascular surface and are suited to investigating endothelial uptake, barrier crossing, and leukocyte migration (Gu et al., 2023; Gaume et al., 2024). Intramuscular or maxillofacial inoculation places virus close to peripheral nerve endings and permits analysis of subsequent neural spread (Chen et al., 2007; Tan et al., 2014). Direct intracerebral inoculation bypasses the initial CNS entry process and instead examines viral replication and spread after exposure of CNS tissue (Hamidi et al., 2025). These approaches establish the feasibility of different mechanisms, but their results cannot be directly compared to determine which entry route predominates during natural infection.

Model dependence is also relevant when interpreting human observations. Histopathology and neuroimaging identify affected neural structures, but the distribution of lesions does not, by itself, establish the direction of viral movement or the earliest anatomical entry site (Xing et al., 2016; Li et al., 2019). More informative comparisons of candidate routes should therefore be conducted, where feasible, within the same validated peripheral infection model, using matched viral backgrounds, host conditions, and observation windows. Complementary endothelial and neuronal systems can then help identify the cellular mechanisms underlying each route. This division of experimental tasks would reduce the risk of treating findings generated from different starting points as equivalent evidence of route use.

### 6.2 What evidence is needed to distinguish competing transmission hypotheses?

Discriminating between peripheral neural and neurovascular entry requires identification of the earliest sites of viral spread and direct assessment of the consequences of route-specific interventions. For a peripheral neural route, serial tissue sampling in experimentally infected animals should determine whether infectious virus or evidence of replication appears in peripheral neural structures before anatomically connected spinal cord or brainstem regions. Nerve-directed interventions could strengthen this inference, but their effects should be compared with appropriate sham controls and simultaneous measurements of peripheral replication and circulating viral burden. This is essential because reduced CNS infection following an intervention may reflect diminished peripheral exposure rather than selective inhibition of axonal spread. Differences between existing nerve interruption studies also indicate that route dependence must be evaluated within a defined experimental context (Chen et al., 2007; Ohka et al., 2022).

For a neurovascular route, the critical question is whether infectious virus or virus-associated carriers reach CNS tissue from the vascular compartment. Experimental studies have demonstrated crossing by vesicle-associated virus and migration of virus-bearing leukocytes across BBB models (Gu et al., 2023; Gaume et al., 2024). However, viral RNA recovered from whole brain tissue does not necessarily distinguish virus retained within cerebral vessels from material that has entered the parenchyma. In animal studies, vascular perfusion before tissue collection, anatomical localization of viral antigen or RNA, and assays for infectious virus should therefore be combined where feasible. Interventions targeting carrier uptake or migration should also be evaluated alongside measurements of systemic viral burden to determine whether their effects occur at the CNS entry interface rather than earlier in dissemination. Studies of the blood–cerebrospinal fluid barrier should be assessed separately from BBB models, because infection of choroid plexus epithelial cells does not establish passage across brain microvascular endothelium (Hügel et al., 2026).

The timing of barrier dysfunction could further distinguish alternative mechanisms of blood-borne entry. If marked BBB disruption is required for initial entry, increased permeability should precede or coincide with the earliest evidence of CNS infection. If virus-associated carriers can cross a relatively intact barrier, infectious material should be detectable beyond the vascular compartment before substantial permeability changes, and interference with the relevant transport process should reduce early CNS exposure. Existing studies demonstrate early vesicle-associated crossing in experimental BBB systems and inflammatory barrier injury in infected animals, but they do not establish a universal sequence during natural infection (Gu et al., 2023; Zhu et al., 2026).

Clinical studies could complement these experiments through serial collection of mucosal specimens and blood, with separate characterization of cell-free, vesicle-associated, and cell-associated viral fractions where technically feasible. Viral RNA detection should be distinguished from demonstration of infectivity. These measurements could be integrated with neurological assessments, neuroimaging, and cerebrospinal fluid findings obtained when clinically indicated. Such observations may constrain the timing of competing transmission hypotheses, although temporal associations alone cannot identify the anatomical route of CNS entry.

### 6.3 How can the multistage progression-constraint model be tested?

The multistage progression-constraint framework can be tested by examining whether the biological processes represented by adjacent stages can be distinguished temporally or through intervention. In clinical cohorts, repeated measurements of peripheral viral burden, circulating viral RNA and its associated fractions, neurological involvement, and indicators of neural or vascular injury could help identify the earliest measurable divergence between uncomplicated and neurological disease. Because some stages may be brief or difficult to sample, a negative result at one time point should not be interpreted as proof that the corresponding process did not occur.

Experimental studies can provide more direct evidence by assessing several processes within the same peripheral infection model. At defined time points, matched groups of animals could be examined for peripheral viral replication, circulating infectious virus, peripheral neural involvement, initial CNS localization, productive CNS infection, and subsequent neural or vascular injury. Viral RNA measurements should be supplemented, where possible, by infectious virus assays and tissue-based evidence of replication. This would help distinguish exposure of CNS tissue from establishment of productive infection and prevent later tissue damage from being used retrospectively as evidence of the initial entry route.

Timed interventions offer a complementary test of stage specificity. Early administration of hyperimmune serum reduced cranial nerve and brainstem infection in a mouse model, demonstrating that intervention near the beginning of infection can alter subsequent neurological involvement (Tan et al., 2014). Other experimental studies have identified TLR7–IL-6, HMGB1, and C5a–C5aR1-associated pathways as contributors to neural or vascular injury (Luo et al., 2019; You et al., 2023; Zhu et al., 2026). Future studies could administer interventions before or after documented CNS localization and measure their effects on circulating viral burden, CNS infectious virus levels, BBB permeability, and tissue injury. A reduction in inflammatory injury without a corresponding change in preceding viral dissemination would support partial separation of these processes. Conversely, effects across several stages would indicate that the targeted pathway has broader functions rather than necessarily invalidating the model.

The framework must also remain open to revision. If sufficiently resolved longitudinal measurements and appropriately controlled interventions repeatedly fail to distinguish the proposed transitions, or reveal patterns inconsistent with their proposed boundaries, the division into five progression constraints should be reconsidered. Such conclusions would require validated stage-specific measurements, since insufficient sampling or assay sensitivity could otherwise conceal genuine differences. The model is therefore useful only insofar as it generates distinctions that can be examined and revised experimentally.

### 6.4 Major limitations of the current evidence base

The principal limitation is the absence of longitudinal evidence reconstructing the full sequence from peripheral EV-A71 infection to neurological injury within the same naturally infected children. Clinical sampling often captures only selected points in the disease course, while pathological specimens predominantly represent advanced infection. Moreover, a negative cerebrospinal fluid viral nucleic acid result does not exclude EV-A71-associated CNS involvement, as virus-specific intrathecal immune responses have been detected in some patients despite negative CSF nucleic acid findings (Leon et al., 2020).

Clinical measurements also have limited capacity to identify the anatomical route of entry. Detection of viral RNA in blood, cerebrospinal fluid, or CNS-associated specimens does not independently establish whether virus crossed the BBB, used the blood–cerebrospinal fluid barrier, or reached neural tissue through peripheral nerves. Experimental approaches can address individual mechanisms more directly, but their results still require validation in the context of natural infection. Consequently, the relative contributions of the proposed routes and the temporal relationships among all five stages remain unresolved in children.

## 7 Conclusion

EV-A71 neurological disease involves mechanistically distinct processes, including CNS access, establishment of productive infection, and subsequent neural and vascular injury. Animal studies provide evidence for peripheral neural spread, whereas CNS barrier models demonstrate mechanisms of blood-borne access, including vesicle-associated transport and migration of virus-bearing leukocytes. Their relative contributions during natural infection in children remain unresolved. Importantly, CNS exposure does not necessarily result in productive infection, and BBB dysfunction observed during severe disease cannot be assumed to precede the initial entry event. The multistage progression-constraint model integrates these distinctions without imposing a fixed entry route or an obligatory sequence of disease progression.

Two research priorities follow. First, where feasible, candidate CNS entry routes should be compared within the same validated peripheral infection model, using matched viral backgrounds, serial anatomical localization, and infectious virus measurements at defined time points. Route-directed interventions should be assessed alongside peripheral replication and circulating viral burden to distinguish inhibition of CNS entry from reduced upstream infection. Second, longitudinal measurements of peripheral dissemination, productive CNS infection, and neural or BBB injury should be combined with appropriately timed interventions to determine whether these processes can be experimentally distinguished. Complementary clinical observations could help establish when their measurable indicators diverge during disease progression. Together, these approaches would allow the proposed model to be tested, refined, or rejected according to the evidence.
